# Advances in Bacterial Lysate Immunotherapy for Infectious Diseases and Cancer

**DOI:** 10.1155/2024/4312908

**Published:** 2024-06-12

**Authors:** Md. Mijanur Rahman, I. Darren Grice, Glen C. Ulett, Ming Q. Wei

**Affiliations:** ^1^ School of Pharmacy and Medical Sciences Griffith University, Gold Coast 4222, QLD, Australia; ^2^ Menzies Health Institute Queensland Griffith University, Gold Coast 4222, QLD, Australia; ^3^ Institute for Glycomics Griffith University, Gold Coast 4222, QLD, Australia

## Abstract

Antigenic cell fragments, pathogen-associated molecular patterns, and other immunostimulants in bacterial lysates or extracts may induce local and systemic immune responses in specific and nonspecific paradigms. Based on current knowledge, this review aimed to determine whether bacterial lysate has comparable functions in infectious diseases and cancer treatment. In infectious diseases, including respiratory and urinary tract infections, immune system activation by bacterial lysate can identify and combat pathogens. Commercially available bacterial lysates, including OM-85, Ismigen, Lantigen B, and LW 50020, were effective in children and adults in treating respiratory tract infections, chronic obstructive pulmonary disease, rhinitis, and rhinosinusitis with varying degrees of success. Moreover, OM-89, Uromune, Urovac, Urivac, and ExPEC4V showed therapeutic benefits in controlling urinary tract infections in adults, especially women. Bacterial lysate-based therapeutics are safe, well-tolerated, and have few side effects, making them a good alternative for infectious disease management. Furthermore, a nonspecific immunomodulation by bacterial lysates may stimulate innate immunity, benefiting cancer treatment. “Coley's vaccine” has been used to treat sarcomas, carcinomas, lymphomas, melanomas, and myelomas with varying outcomes. Later, several similar bacterial lysate-based therapeutics have been developed to treat cancers, including bladder cancer, non-small cell lung cancer, and myeloma; among them, BCG for in situ bladder cancer is well-known. Proinflammatory cytokines, including IL-1, IL-6, IL-12, and TNF-*α*, may activate bacterial antigen-specific adaptive responses that could restore tumor antigen recognition and response by tumor-specific type 1 helper cells and cytotoxic T cells; therefore, bacterial lysates are worth investigating as a vaccination adjuvants or add-on therapies for several cancers.

## 1. Introduction

Bacterial lysates (BLs) or extract-based preparations comprising antigen-rich cell fragments, pathogen-associated molecular patterns (PAMPs), and several other immunostimulants stimulate the immune system to recognize and combat infections [1, 2, 3, 4, 5]. It has been claimed that such preparations induce local and systemic immunomodulation, but clinical trials have found conflicting results. BLs are widely used therapeutically, though their immune system effects are only partially known [6]. Polyvalent mechanical bacterial lysates (PMBLs) and polyvalent chemical bacterial lysates (PCBLs) are two types of BLs used in clinical settings [7]. Patients with recurring respiratory tract infections (RTIs) and children with otitis media or nasopharyngitis benefit more from PMBLs than PCBLs [8]. This may be because PMBLs contain comparatively less damaged and more chemically pure bacterial antigens than PCBLs, increasing immunogenicity [9].

Since the 1950s, BLs have been used to reduce the incidence of recurrent respiratory tract infections (RRTIs) in children and adults [7]. Some commercially marketed BLs are beneficial in treating RTIs, chronic obstructive pulmonary disease (COPD), rhinitis, rhinosinusitis, and urinary tract infections (UTIs). In pediatric RTIs, BLs lowered the incidence rate (OM-85, Luivac, and Ismigen), shortened the duration (OM-85 and Luivac), decreased the frequency of acute episodes (Lantigen B), and reduced the need for antibiotics (OM-85 and Luivac). Luivac lowered the incidence and duration of RTIs in adults, limited the need for antibiotics, and decreased the bacterial count in colonized individuals [8, 10, 11, 12, 13]. Moreover, BLs lowered the severity (Ismigen) and frequency (OM-85, Ismigen) of exacerbations and the number of days with fever and hospitalization (Ismigen) in adults with COPD [14, 15]. In pediatric rhinosinusitis, OM-85 lowered the incidence of rhinosinusitis attacks, and Ismigen decreased the intensity of allergic rhinitis (AR) symptoms. Additionally, OM-85 reduced the incidence of RTIs and antibiotic usage in HIV + individuals. In adult UTIs, OM-89 (Uro-Vaxom) reduced the frequency of recurrences and lowered the incidence of bacteriuria, dysuria, and leukocyturia. In pediatric UTIs, Urivac proved effective in generating relapse-free progression of recurrent UTIs [15, 16, 17].

A meta-analysis of 19 studies comparing BLs treatments to a control group found a 24% improvement in allergy symptoms and also improved asthma symptoms by 22% and rhinitis symptoms by 300% more than the control group [18]. In a noninterventional prospective cohort study of 57 children with repeated otitis media (middle ear infections) or pharyngotonsillitis episodes in Spain, a 3-month treatment with bacterial immunostimulant combinations made from bacterial lysates (either polyvalent-specific vaccines (made from the lysate of *Streptococcus pneumoniae*, *Haemophilus influenzae*, *Streptococcus pyogenes*, and *Moraxella catarrhalis*) or auto-vaccines) reduced the frequency, severity, and consequences of acute episodes after 6 months [19]. Another meta-analysis of five trials found that using BLs (PCBLs: OM-85; PMBLs: Ismigen, MV130, and MV310) reduced wheeze episodes and asthma exacerbations [20]. Moreover, the BLs group demonstrated improving AR symptoms three times faster than the control group in a meta-analysis of three studies [21, 22, 23].

Furthermore, a systematic review and meta-analysis revealed that BLs such as Uro-Vaxom®, Urovac®, and ExPEC4V reduced UTI recurrence compared to placebo. Uro-Vaxom reduced UTI recurrence the most among the BLs, with a greater benefit at 3 months compared to 6 months after therapy. Urovac treatment also indicated a decrease in the recurrence of UTIs [24].

William Coley developed “Coley toxins” in 1891, a heat-killed mix of *S. pyogenes* and *Serratia marcescens*. Patients with sarcomas, carcinomas, lymphomas, melanomas, and myelomas treated with them with varying degrees of success [4, 25, 26]. Multiple months (precisely 4 months) of treatment with “Coley toxins” led to complete tumor remission in an inoperable sarcoma case [27]. The 5-year survival rate for inoperable carcinomas was 34%–73% and for sarcomas 13%–79%, depending on the tumor subtype. Coley's therapy (preparation methods of the “Coley toxins” were inconsistent) results were ambiguous, unreplicable, and unpredictable; consequently, new radiation therapy replaced it in the early twentieth century. As immunology progressed, “Coley toxins” were found to stimulate and regulate the immune system in a multilevel manner—this increased interest in the medicinal potentials of Coley's approach [28]. Later, several bacterial lysate- or extract-based immunotherapies were developed, including intravesical bacillus Calmette–Guérin (BCG) for in situ bladder carcinoma [29, 30, 31], heat-killed *Mycobacterium indicus pranii* for invasive bladder cancer and non-small cell lung cancers (NSCLC) [32, 33], and heat-killed whole-cell *Mycobacterium obuense* for melanoma patients [34].

## 2. How Does Bacterial Lysate-Based Immunotherapy Work in General?

Numerous research data now exist showing that BLs function as immunomodulators capable of eliciting antibodies against pathogens and producing immunoregulatory responses in mucosal tissues [8, 17, 35, 36, 37, 38]. Notably, they may interact with diverse cells—such as monocytes or macrophages (M*φ*), dendritic cells (DCs), or epithelial cells—through the engagement of surface Toll-like receptors (TLRs) with bacterial wall components, such as proteoglycans or lipopolysaccharides (LPS). These interactions drive the development of monocytes into M*φ* and activate immature DCs, producing specific chemokines and cytokines [6].

Activation of both nonspecific and specific immunological pathways is required for bacterial lysates to exert their immune impact. Activation of nonspecific mechanisms (innate immune system) involves signal transmission by TLRs (TLR-2/TLR-6, TLR-9, and TLR-7/TLR-8), an increase in chemotaxis as well as cytotoxic and phage activity of phagocytes (M*φ*, DCs, and neutrophils), an increase in the activity of natural killer (NK) cells (CCL2, 3), activation of DCs along with a proliferation in their antigen presentation ability and increased migration into the respiratory tract, activation of specific mechanisms (acquired immune system) is marked by an increased concentration of immunoglobulins (Ig) A and IgG antibodies, increased T-cell activity and the ability to activate other specific mechanisms (activation of T and B cells), activation of B cells (CCL2, CCL3, CCL20, CCL22, BAFF, IL-6, and APRIL), and increased activity of CD4+ CD25+ Foxp3+ regulatory T cells [39].

## 3. Successful Immunotherapeutic Bacterial Lysates for Infectious Diseases

### 3.1. OM-85

OM-85 (Broncho-Vaxom®, Broncho-Munal®, Ommunal®, Paxoral®, and Vaxoral®) is an over-the-counter oral preparation of lyophilized bacterial lysates of eight common respiratory pathogens (*H. influenzae*, *Diplococcus pneumoniae*, *Klebsiella pneumoniae*, *Klebsiella ozaenae*, *Staphylococcus aureus*, *S. pyogenes*, *Streptococcus viridans*, and *Neisseria catarrhalis*). OM-85 has been used for several decades in different countries (excluding the USA as OM-85 or similar products are not yet approved by the FDA) in both adults and children to treat and prevent recurrent respiratory infections, including COPD, asthma, and rhinosinusitis [17, 40]. However, in a review in 2019, the European Medicines Agency recommended the use of BLs only for the prevention of RRTIs except for pneumonia, and they also stated that there are no adequate data to support that the BLs are effective in treating existing respiratory infections or preventing pneumonia [41]. OM-85 was shown to have immunomodulatory [36] and antiviral [42, 43] properties and is widely used to prevent recurrent upper respiratory infections in adults and children [44, 45], with an excellent safety profile [10, 44]. In addition, OM-85 decreased the frequency and duration of wheezing episodes in preschool children with acute respiratory infections [46]. It slowed the onset of severe lower respiratory diseases in infants at risk [47]. Because wheezing-inducing lower respiratory tract infections are common precursors of childhood asthma [48, 49], an NIH-sponsored trial (NCT02148796) is currently testing whether OM-85 given to high-risk, 6–18 months infants for 10 days, monthly, for 2 consecutive years can prevent or delay the development of wheezing or asthma-like symptoms during a 3-year observation period off therapy in these young children [50].

OM-85 also reduced RTIs and exacerbations in patients with allergic rhinitis, asthma, or COPD [51]. After treatment with OM-85, RTIs decreased by 45% (38/69, *p*  < 0.05). OM-85 with standard optimized care reduced medical exacerbations by 36%. In addition, OM-85 increased serum and secretory IgA levels. Its safety profile was also acceptable, with only minor gastrointestinal (nausea, vomiting, and stomach discomfort) and dermatologic (rash and urticarial) side effects in clinical studies [17].

In Ukraine, OM-85 was tested for clinical and immunological efficacy in children with RTIs. Acute RTIs were found in 108 children aged 1–3 years: 57 received conventional treatment, and 51 got OM-85 at 3.5 mg daily. Overall weakness decreased by 0.99 days, cough by 1.45 days, and catarrhal manifestations 1.23 days earlier in the OM-85-treated group (*p*  < 0.05). OM-85 therapy supplementations also normalized phagocytic levels, CD3+, CD4+, and CD21+ cell concentrations along with IgA and IgM levels [52].

Esposito et al. [53] conducted a prospective, randomized, single-blind study to compare the immune response, efficacy, and safety of conventional inactivated influenza vaccines IIV (Fluarix, GlaxoSmithKlineBiologicals, Rixensart, Belgium) in 68 children with RRTIs who were vaccinated with (*n* = 33) or without (*n* = 35) OM-85. Respiratory morbidity significantly decreased in children treated with OM-85 (*p*  < 0.05), positively impacting RRTI incidence. The two groups had similar adverse effects. These findings show that OM-85 does not affect children's influenza vaccine immunological response. However, OM-85 before and during the IIV vaccine reduces respiratory morbidity and is well-tolerated and safe [53]. A meta-analysis of OM-85 in pediatric RRTIs found that it reduced respiratory infection frequency (mean difference, MD = −2.22 days, 95% CI (−2.75, −1.90)) and wheezing duration (MD = −3.37 days, 96% CI (−4.52, −2.22)) [54]. Two other meta-analyses found that OM-85 prevented respiratory infection in children but did not address wheezing or allergic illness [55, 56]. Another meta-analysis of 53 trials revealed that OM-85 reduced respiratory infections in children with RRTIs [13]. Moreover, OM-85, coupled with standard asthma medication, may prevent RRTIs and decline asthma attack intensity in children. Sixty asthmatic children were placed into two groups: OM-85 (orally) (24 children, 15 boys, and 9 girls aged 5–14 years) or conventional inhaled corticosteroid (ICS) (36 children, 23 boys, and 13 girls aged 5–15 years). Both groups had considerably fewer asthma episodes after treatment. After OM-85 treatment, peripheral blood NKT and CD4+ NKT cells increased and had higher levels of IFN-*γ*, IL-4, and IL-10, whereas IL-4 levels were lower than the ICS group [57].

Clinical investigations also showed that OM-85 reduces acute COPD exacerbations, antibiotic prescriptions, and symptoms. A randomized, double-blind, placebo-controlled clinical study by Tang et al. [58] examined the efficacy and safety of OM-85 in Chinese adults with COPD or chronic bronchitis. OM-85 significantly reduced the proportion of COPD patients with recurrent acute exacerbations (defined as two or more exacerbations) compared to placebo during the 12-week treatment period, and these benefits were maintained during the 10-week follow-up. The results support earlier studies of OM-85 in Asian and Caucasian populations, showing that it protects against chronic bronchitis and COPD exacerbations [58]. A meta-analysis of 13 RCTs found that OM-85 significantly reduced COPD exacerbations (*p*  < 0.01; weighted mean difference (WMD) = −0.86; 95% CI (−1.38, −0.34)) and antibiotic treatment days (*p*  < 0.01; WMD = −9.49; 95% CI (−11.93, −7.05)). The negative cost-effectiveness ratio also favors OM-85 therapy [59].

According to a double-blind RCT [60], oral OM-85 has outstanding and sustained efficacy in treating AR symptoms and may be an alternate treatment for persistent AR. OM-85 therapy elevated IFN-*γ* and reduced IL-4 and IL-13 in nasal lavage. Reduced scores for overall nasal symptoms, itching, rhinorrhea, and sneezing (*p*  < 0.05) were also found. Eosinophils in nasal smears decreased following OM-85 therapy. The OM-85-induced improvements lasted 4 to 8 weeks. In chronic rhinosinusitis (CRS), OM-85 reduced the number of episodes and days with antibiotics per month in children and provided long-term prevention [22]. The European Position Paper on rhinosinusitis and nasal polyps supports OM-85 for CRS therapy in adults with level 1b evidence [61].

For AD, a study found that OM-85 reduced recurring episodes by 20% compared to placebo. Oral delivery of OM-85 to children with established AD substantially reduced and delayed new flares (hazard ratio (HR) of recurrent new flares = 0.80; 95% CI (−0.29, 1.63)). OM-85 and placebo had similar and excellent tolerance with no severe adverse effects [62]. In contrast, a randomized placebo-controlled study of 606 neonates found no difference in AD prevalence between the bacterial lysate and placebo groups. However, early bacterial lysate feeding may prevent AD in babies with a single atopic family background [54].

Bacterial antigen therapy for allergic illness is still debated. BL therapy may lessen asthma attack length, coughing, and wheezing compared to the control group [57, 63], while other studies have shown no significant difference in allergy episodes between treated and placebo groups [64]. Interestingly, Pivniouk et al. [65] found that providing OM-85 to the airway compartment protects against experimental allergic asthma by activating several immune pathways, and this protection needed a cumulative dosage 27- to 46-fold lower than the oral route. Intranasal OM-85 may be more effective because it directly accesses airway mucosal networks when asthma first develops. Cardinal cellular and molecular asthma characteristics were assessed in mice, sensitized and challenged with ovalbumin or *Alternaria*, and given OM-85 intranasally. In all animals, airway OM-85 controlled allergic asthma by targeting the airway epithelium/IL-33/ILC2 axis, lung allergen-induced type 2 responses, and DCs whose Myd88/Trif-dependent tolerogenic reprogramming was enough to prevent asthma. Allergies are often accompanied by an immune system imbalance toward type 2 T helper (Th2) and away from type 1 T helper (Th1) cell response [18]. Several studies [66, 67, 68] found that OM-85 could switch the immunological response from Th1/Th2 to Th1.

According to an *in vitro* study of Pivniouk et al. [69], OM-85 can lower ACE2 expression in human airway cell lines and primary cells. By downregulating SARS-CoV-2 receptor expression, OM-85 suppresses epithelial cell infection *in vitro*. After going through proper clinical trials, OM-85 might be utilized as a preventative medication alongside vaccinations and other future medicines to stop COVID-19 (Table 1).

#### 3.1.1. Mode of Action of OM-85

PAMPs like OM-85 mature mucosal DCs in gut-associated immunological tissue (Peyer's patches through M cells), activating innate and adaptive immune responses. Downstream events include activation of monocytes, NK cells, M*φ*, and T cells such as Th1 cells, regulatory T (Treg) cells, and cytotoxic CD8+ cells; epithelial and mucosa antimicrobial peptide expression; polymorphic neutrophil recruitment to the lung; B-cell and antiviral Th1-related cytokine production; and B-cell maturation (leading to increased serum and airway immunoglobulins). OM-85 also supports immune system development in children by downregulating Th2 and activating Treg cells to restore Th1/Th2 imbalances [60, 91].

Tang et al. [58] suggested OM-85 may stimulate lung macrophage phagocytic activity, DC function, and respiratory tract secretory IgA (sIgA) production. Sinonasal immunity studies have shown that bitter taste receptors (T2R) enhance ciliary beat frequency (CBF) and other local immune defenses such as nitric oxide (NO) release and antibacterial chemical production. Air–liquid interfaces (ALIs) were pretreated with nitric oxide synthase (NOS) or T2R signal transduction inhibitors to test whether topical bacterial lysate works by a comparable mechanism. A study discovered that blocking OM-85-induced CBF and NO generation revealed that the observed effects are NO-dependent, transient receptor potential cation channel subfamily M member 5 (TRPM5)-dependent, and phospholipase C isoform *β*2 (PLC*β*2)-dependent, indicating T2R activation. This shows that topical OM-85 may directly activate T2R and the immune system in response to the bacterial antigens in the lysate, unlike oral treatment [17].

Some evidence showed that OM-85 stimulates TLRs. Host pattern recognition receptors (PRRs), most notably TLRs, recognize conserved PAMPs for innate immunological signaling [92]. Without myeloid differentiation, primary response 88 (MyD88) protein, OM-85 could not establish influenza immunity in mice. However, treating with TLR2/6 and TLR9 ligands simultaneously protects against pathogenic gram-positive and gram-negative bacteria [92]. This protective effect in isolated lung epithelial cells was accompanied by fast pathogen death in the lungs. Other findings indicate that OM-85 may trigger TLR4 and TLR2 [93]. It also boosts Treg cell growth and Th1 cell responsiveness [94]. *In vitro*, OM-85 preferentially activated nuclear factor kappa-light-chain-enhancer of activated B cells (NF-kB) and mitogen-activated protein kinase (MAPK) in human monocyte-derived DCs from COPD patients [95]. DCs and peripheral blood mononuclear cells (PBMC) significantly upregulated chemokines (CXCL8, CXCL6, CCL3, CCL20, and CCL22) and B-cell-activating cytokines (IL-6, BAFF, and IL-10). In asthma and COPD, OM-85 remarkably reduced rhinovirus-induced cell death and virus multiplication in human bronchial epithelial cells [43]. OM-85 appreciably boosted virus-interacting proteins C1q-R and *β*-defensin expression, relevant for antigen presentation and phagocytosis while reducing rhinovirus-induced intracellular adhesion molecule 1 (ICAM-1) expression [96].

Polyclonal B-cell activation by OM-85 increased serum IgG and bronchoalveolar lavage IgA and IgG. The binding and neutralization of influenza virus by IgA protects against associated bacterial infections. These findings imply that OM-85 induces polyclonal mucosal antibody responses and increases adaptive CD8+ T-cell responses. OM-85 protected mice against nebulized influenza viral infection by increasing IL-6 and tumor necrosis factor *α* (TNF-*α*) expression in bronchoalveolar lavage fluid compared to fatal influenza infection [97]. Boosted inflammation decreased virus titers and host mortality [98].

**Figure 1:**
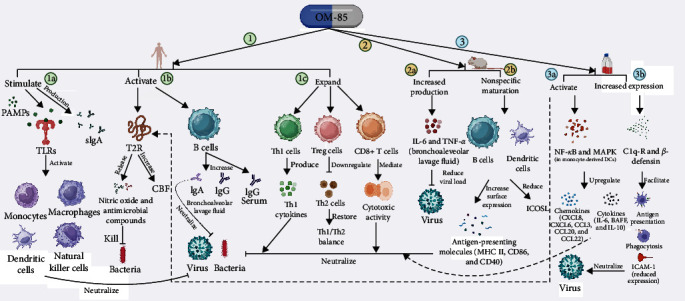
Mechanisms of action of the most studied bacterial lysate OM-85. In humans, pathogen-associated molecular patterns (PAMPs) of OM-85 stimulate the Toll-like receptors of the immune cells and activate monocytes, macrophages, dendritic cells (DCs), and natural killer cells, activating the immune system to fight infections. OM-85 also stimulates the production of secretory immunoglobulin A (sIgA) (1a). In sinonasal immunity, OM-85 activates bitter taste receptors (T2R), resulting in increases in ciliary beat frequency (CBF) (indicating improved lung functions), and other local immune defenses such as the release of nitric oxide (NO) and secretion of antimicrobial compounds lead to the killing of pathogens. Moreover, OM-85 also activates polyclonal B cells, increasing IgG levels in serum and IgA and IgG levels in bronchoalveolar lavage. IgA neutralizes viruses and controls secondary bacterial infections (1b). An effect on the expansion of type 1 helper T (Th1) cells, regulatory T cells (Treg), and CD8+ T cells has also been observed with the administration of OM-85. Th1 cells produce inflammatory cytokines, which send signals to immune cells to destroy pathogens, whereas the cytotoxic activity of CD8+ T cells also does the same. Treg cells downregulate the Th2 cells and restore Th/Th1 balance, improving overall mucosal immunity (1c). In a mice study, OM-85 increased the production of IL-6 and TNF-*α* in bronchoalveolar lavage fluid, contributing to reduced viral load (2a). Also, nonspecific maturation of B cells and DCs by OM-85 leads to increased surface expression of antigen-presenting molecules (MHC II, CD86, and CD40) and helps to neutralize pathogens, and a decrease in inducible T-cell costimulator ligand (ICOSL) provides protection against allergic responses (2b). *In vitro*, data showed that OM-85 could activate T2R (downstream processes are like in human studies) and NF-kB and MAPK in monocyte-derived DCs from COPD patients, resulting in the upregulation of chemokines (CXCL8, CXCL6, CCL3, CCL20, and CCL22), and B-cell-activating cytokines (IL-6, BAFF, and IL-10) may impact in fighting against pathogens (3a). OM-85 also increased the expression of virus-interacting proteins such as C1q-R and *β*-defensin, essential for antigen presentation and phagocytosis, and significantly reduced rhinovirus-induced expression of intracellular adhesion molecule (ICAM)-1, resulting reduced viral load (3b).

Furthermore, OM-85 oral treatment for 10 days in BALB/c mice increased influenza virus immune response, reduced viral load, and susceptibility to *S. pneumoniae* secondary infections [42]. The maturation of DCs and B cells was nonspecific, with increased surface expression of antigen presentation molecules (MHC II, CD86, and CD40) and decreased inducible T-cell costimulator ligand (ICOSL). ICOSL–ICOS interactions cause allergic Th2 response, whereas OM-85 protects against it. The balanced release of cytokines and anti-inflammatory activity factors by OM-85 creates an “early warning” state that protects against infectious pathogens and controls inflammation. The three activation routes were rebalancing Th1/Th2, activating B cells via a T-cell-independent mechanism, releasing chemokines, and activating polymorphonuclear leukocytes [96] (Figure 1).

### 3.2. Ismigen

Ismigen, a PMBLs immunomodulator, contains *S. aureus*, *S. pyogenes*, *S. viridans*, *S. pneumoniae* (six different serotypes: TY I/EQII, TY2/EQ22, TY3/EQ 14, TY5/EQ 15, TY8/EQ23, and TY47/EQ24), *K. pneumoniae*, *K. ozaenae*, *H. influenzae* serotype B, and *M. catharralis* [72]. Mechanical lysis yields ismigen, which has benefits over chemical lysates as immunomodulators. With mechanical lysis, corpuscular antigens are better retained, allowing for a long-term immune response. Mechanical lysates were significantly more effective than chemical lysates in treating lower RTIs with Imigen/Respibron® [99].

In grass pollen-sensitive children, sublingual Ismigen reduced seasonal AR symptoms [15]. Ismigen may impair Th2 cell response by affecting mucosal immunity. In a study, the Ismigen-administered group showed a significant decrease in total nasal symptom (*p*=0.001), total ocular symptom (*p*=0.04), and visual analog scale scores for nasal and eye symptoms (*p* < 0.001 and *p* < 0.001, respectively), as well as an increase in peak nasal inspiratory flow (*p*=0.04) compared to the placebo group. The placebo group had a statistically significant rise in allergen-specific IgE concentrations, whereas the Ismigen group did not [15]. However, a double-blind, placebo-controlled randomized trial of 80 children between 5 and 17 years showed that around one of the two children with seasonal AR has been found with nasal carrier of *S. aureus*. The nasal colonization of *S. aureus* has not been affected by the sublingual treatment of Ismigen, and thus, Ismigen was not recommended to remove *S. aureus* from the nasal carrier [70]. In another trial, Ismigen reduced AR symptom severity in 61.5% of patients [21].

A double-blind, randomized, placebo-controlled study found that Ismigen medication significantly reduced exacerbation frequency (215 vs. 248 instances), length (10.6 vs. 15.8 days), antibiotic usage (−270 doses), and hospitalization time (275 vs. 590 days) in moderate-to-very severe COPD for study vs. control. Two groups of 178 adults were randomized to receive Ismigen (first 10 days each month for 3 months) or a placebo. After therapy, patients were monitored for 9 months. Selected clinical outcomes were considerably lower in the lysate group than in the placebo group. Even in severe COPD patients with comorbidities, Ismigen prophylaxis reduces acute exacerbation episode frequency, intensity, and duration [14].

Hans et al. [72] found that Ismigen selectively inhibits the most prevalent respiratory pathogens (*S. aureus*, *S. pyogenes*, *K. pneumoniae*, *H. influenzae*, and *M. catarrhalis*), restoring the oropharynx's eubiotic colonization profile 1–2 months after therapy.

A meta-analysis of 15 randomized clinical trials in 2,557 individuals found that PMBLs reduce respiratory tract infection recurrence in children and adults (relative risk: −0.513, 95% CI (−0.722, −0.303), *p* ≤ 0.001) [8]. Prospective open research examined whether a PMBL treatment (drug name not stated in the abstract) might induce an efficient and well-targeted immune response and improve clinical outcomes. Twenty-five of 27 clinically responding individuals had increased salivary PMBLs antigen-specific antibodies. The link between PMBL-specific immunoglobulin titers and clinical findings was significant for IgG and IgA but not IgM. Only patients with clinical improvement showed Th1 switch, whereas three responders and four nonresponders showed Th0. One clinically responding patient had weak Th2 polarization. Samples from responder patients showed opsonization of live microorganisms [35]. In addition, Avdeev et al. [100] found that mechanical bacterial lysate (MBL) may prevent severe COPD exacerbations. Frequent COPD exacerbations were studied in 60 individuals (groups C and D according to the GOLD classification) and blindly assigned into two groups. After 6 months, the MBL group showed statistically significant improvements in respiratory function, exacerbation frequency, emergency medical service use, basic therapy adjustments, and hospitalization. MBL therapy was safe and had little side effects. On the contrary, a phase IV, randomized, controlled, double-blind, multicenter Advanced Immunological Approach in COPD Exacerbation (AIACE) trial included 142 adult patients in the placebo group and 146 in the treatment group failed to meet its primary endpoint—a reduction in exacerbations of COPD [73]. In 49 children (21 received Ismigen and 28 received a placebo) with allergic asthma and dust mite allergy, a double-blind, placebo-controlled, multicenter, randomized trial conducted in Poland revealed that Ismigen may have a preventative impact on episodes of asthma exacerbation connected to infections as well as those unrelated to them [71] (Table 1).

### 3.3. Lantigen B

Lantigen B is a chemically lysed preparation of six respiratory tract bacteria (*S. aureus*, *K. pneumoniae*, *S. pneumoniae*, *S. pyogenes*, *M. catarrhalis*, and *H. influenzae*). Since its development in the 1960s, Lantigen B has been experimentally used to reduce winter RTIs [11].

A double-blind, placebo-controlled, multicenter clinical study found that Lantigen B reduced infectious episodes in recurrent RTIs. The placebo group had a mean of 1.43 (95% CI (1.01, 1.86)) episodes throughout the 8-month trial, whereas the Lantigen B-treated group had 0.86 (95% CI (0.54, 1.19)). The treated group used antibiotics for 1.24 days compared to 2.83 in the placebo group [64].

More recently, a therapeutic dosage of Lantigen B was found to alter the primary SARS-CoV-2 receptor in oropharyngeal cells and lower viral production *in vitro*. This action may work synergistically with vaccination and treatment to reduce the number of potentially infected cells and, thus, lower SARS-CoV-2 infection [11] (Table 1).

### 3.4. LW 50020 (Luivac)

LW 50020 (Luivac) is a chemically lysed preparation, an oral immunomodulator containing the antigens of seven RTI-causing bacteria (*S. aureus*, *Streptococcus mitis*, *S. pyogenes*, *S. pneumoniae*, *K. pneumoniae*, *M. catarrhalis*, and *H. influenzae*), and induces specific and nonspecific immune responses in mucosa-associated lymphoid tissue [12, 75]. Several controlled clinical investigations showed that LW 50020 lowers RTIs in children and adults [12, 101, 102].

A multicenter, open study in 14 countries in Europe, Latin America, and Asia enrolled 4,965 individuals with recurrent RTIs. They found that LW 50020 is safe and effectively reduces the incidence, severity, duration, and socioeconomic consequences of ubiquitous RTIs. Compared to the first trial period (first course of LW 50020 and drug-free interval), in the second trial period (second course and follow-up) with RTIs, antibiotic and symptomatic treatments and school or work absences decreased by at least 50%. Excellent or very good tolerability and acceptance were reported by 99% of the research participants [75].

Recurrent RTIs have been treated with oral LW 50020 in a prospective, placebo-controlled, randomized, double-blind trial. The trial found that bacterial lysate-receiving patients had a much lower clinical severity score than placebo patients. No primary medication adverse effects were reported [12].

An open-label, noncomparative Thai trial found that oral LW50020 was safe and effective in protecting patients from RTIs with or without risk factors. Other clinical benefits were reduced infection severity, duration, treatment expense, and school or work absences. After the research, RTIs per month were 63.5% lower than in the 12 months before. Tolerability and acceptability were excellent and acceptable in 96.8% of patients [74] (Table 1).

### 3.5. Polyvaccinium Mite

Intranasal polyvaccinium mite (a PCBL consisting of *S. aureus*, *Staphylococcus epidermidis*, *Streptococcus salivarius*, *S. pneumoniae*, *S. pyogenes*, *Escherichia coli*, *K. pneumoniae*, *H. influenzae*, *Corynebacterium pseudodiphtheriticum*, and *M. catarrhalis*) added to standard antiallergy treatment during grass pollen season does not improve the process AR in children but significantly increased peak nasal inspiratory flow (*p*=0.01) and decreased nasal symptoms on the visual analog scale (*p*=0.03) compared to placebo [76] (Table 1).

### 3.6. OM-89 (Uro-Vaxom)

OM-89 (Uro-Vaxom) contains bacterial extracts from 18 uropathogenic *E. coli* strains. The European Association of Urology (EAU) recommends this immune-prophylaxis medication for women with recurrent uncomplicated UTIs [103]. It stimulates innate and adaptive immunological responses by stimulating DCs and Treg cells, promoting cellular migration to the urothelial mucosa [104].

A 2005 global, randomized, double-blinded, placebo-controlled study found that OM-89 significantly reduced UTIs. There were 453 individuals treated, 231 with OM-89, and 222 received a placebo. The OM-89 treatment substantially reduced postbaseline UTI rates by 34% compared to the placebo group (0.84 vs. 1.28; *p*  < 0.003) [78]. Moreover, 62 women with recurrent UTIs in weeks 16–28 of pregnancy received one OM-89 capsule daily until delivery in an open-label, multicenter pilot study. Only 19.4% of women had UTIs throughout the trial, compared to 52.5% in the 6 months before. Urinary tract infections were considerably decreased following OM-89 prophylaxis (*p*=0.001). In addition, dysuria improved, and antibiotic use reduced. Only two patients had minor adverse effects (nausea and heartburn), and all newborns were healthy [79]. In a 6-month, double-blind cross-over trial of 70 paraplegic individuals, OM-89 drastically reduced UTI recurrence compared to placebo [82]. Rugendorff [81] found excellent tolerability and a statistically significant reduction in reinfection in lower UTI patients. A randomized, double-blind experiment found a substantial decrease in UTI recurrences (*p*  < 0.0005) in the OM-89 group compared to the placebo group, and 67.2% of patients had no repeats (*p*  < 0.0005). The incidence of bacteriuria, dysuria, and leukocyturia decreased considerably. No adverse effects were reported with OM-89 in the trial [80]. Moreover, Tammen and Frey [83] found that treating UTIs with OM-89 considerably decreased recurrences throughout the trial period compared to the pretrial period (*p*  < 0.001). The product was well tolerated: 4.4% of 521 patients reported adverse symptoms, and just two discontinued therapy (0.4%).

Another randomized double-blinded placebo-controlled trial of 451 patients found no meaningful difference in UTI reduction; however, the study's UTI rates were low, which may have impacted the findings [105]. A retrospective medical record assessment [77] found that OM-89 was beneficial for treating recurrent cystitis in women with uncontrolled diabetes (Table 1).

### 3.7. Uromune®

The sublingual vaccination, Uromune®, a mucosal immunostimulant, contains equal amounts of inactivated whole bacteria *E. coli*, *K. pneumoniae*, *Proteus vulgaris*, and *Enterococcus faecalis* [84]. A retrospective cohort analysis of 669 women's medical records by [85] found that Uromune® decreased the risk of recurrent UTIs by 90.28% (87.18%–93.38%) with no adverse effects and lowered antibiotic use. In a multicenter observational trial of 319 women treated with Uromune®, it was found that it significantly reduced recurrent UTIs (*p* < 0.0001) [86]. A UK prospective trial of 77 women indicated that 78% of women who took sublingual Uromune® daily for 3 months did not have another UTIs in the following 12 months [84] (Table 1).

### 3.8. Urovac

Urovac, a whole-cell vaccine, contains heat-killed bacteria from 10 human uropathogenic strains, including six *E. coli* strains and one strain each of *Proteus mirabilis*, *Proteus morganii*, *E. faecalis*, and *K. pneumoniae* in equal proportions. A double-blind, placebo-controlled, phase II clinical study found that women who received six vaccination doses were infection-free longer than those who had placebo or primary immunizations. A total of 55% of women with six vaccinations did not have UTIs, but 89% of placebo-treated women did. The women experienced no severe side effects [88]. A randomized, double-blind, placebo-controlled clinical trial [87] showed that multivalent vaginal mucosal vaccination reduced *E. coli* urinary tract infections. The data implied that sexually active women aged 20–50 may benefit most from the immunization. Another trial by Uehling et al. [89] found that Urovac delayed recurrent UTI reinfection by 8 weeks compared to the placebo. The mean time to reinfection was delayed from 8.7 weeks for placebo-treated women to 13 weeks for vaccine-treated women, with variable immunological responses in serum, urine, and vaginal fluid. There were no significant side effects (Table 1).

### 3.9. Urivac

A prospective, open, multicentered, parallel-group randomized CRUTIL study found that a preventative single dose of urinary antiseptic at bedtime and the hexavalent vaccine Urivac (lysates of *Propionibacterium acnes*, *K. pneumoniae*, *Pseudomonas aeruginosa*, *E. faecalis*, *E. coli*, and *P. mirabilis*) was most effective in preventing recurrent UTIs. The research included 83 recurrent UTI-afflicted children aged 3–15 years. Three groups of children were randomized: 22 received lysate Urivac treatment, 28 received another BLs Uro-Vaxom therapy, and 33 received standard therapy. The first group (Urivac) ended the trial with 87% (19) relapse-free patients. Uro-Vaxom-treated children had 72% (20) relapse-free patients (OR = 2.5; *p* > 0.05, the minimum value of predicted event−4.84), and 17 children at 1-year follow-up. Only 13 children (40%) without nighttime urine antiseptic and BLs had no recurrence (*p* ≤ 0.05, OR = 0.26 with the hexavalent vaccination group) [16] (Table 1).

### 3.10. ExPEC4V

The ExPEC4V candidate vaccine had four bioconjugates with the O-antigens of the four extraintestinal pathogenic *E. coli* serotypes: O1A, O2, O6A, and O25B. A phase I, first-in-human, staggered, randomized, placebo-controlled single-blind trial in 13 Swiss medical centers found that ExPEC4V vaccination reduced UTIs and evoked robust, lasting, and functional immune responses [90] (Table 1).

### 3.11. *E. coli* and *E. faecalis* Lysates

A randomized, double-blind, placebo-controlled clinical trial in Germany with 606 newborns showed that prophylactic treatment with sterile BLs of *E. coli* and *E. faecalis* in infants with single heredity for atopy for 26 weeks in the 1st year of life is cost-effective at 3 and 6 years because it reduced prevalence [106]. However, oral BLs of heat-killed *E. coli* and *E. faecalis* administered in the first 7 months of infancy did not affect AD, asthma, and AR at school age [107].

### 3.12. *Helicobacter pylori* Lysates

The systemic immune response to *H. pylori* cell sonicates was studied in children with symptomatic gastroduodenal illness by Czinn et al. [108]. Patients with positive cultures had considerably greater serum IgA antibody levels to *H. pylori* cell sonicates than controls. Serum IgM antibody levels were not substantially higher in *H. pylori* patients [108]. In another study, *H. pylori*-infected C57BL/6 mice were subsequently inoculated with *H. pylori* lysate either orally with cholera toxin or intraperitoneally with alum using vaccination techniques previously shown prophylactic protection. Oral vaccination raised blood IgG + M and gastric IgA antibodies against *H. pylori* antigen and Th1-specific mucosal CD4+ T-cell proliferation. Parenteral vaccination dramatically increased *H. pylori*-specific serum antibody titers but did not boost mucosal antibody or cellular immune responses compared to control infected animals. Thus, *H. pylori*-specific mucosal immune responses with a Th1 profile may give therapeutic protection [109].

### 3.13. Autologous Bacterial Lysates (ABL)

Autologous bacterial lysates (ABL) are made by inactivating the isolated bacteria from the infected site by heat and homogenizing them in a solution to promote IgG and IgM production and T-cell activation [110]. Ahumada-Cota et al. [111] examined the effects and composition of an ABL for treating and preventing recurrent UTIs in adults. Remission occurs in 70% of patients within 3 months of treatment, and the infection is controlled for 6–12 months. LC-MS examination of patients' sera reveals cytosolic proteins, fimbriae, outer membrane proteins, and LPS in ABL fractions. The ABL treated and controlled recurrent UTIs in adults, and its composition suggests that *E. coli* surface components might be exploited to build a polyvalent protective vaccination. Unlike standard antimicrobials, ABL therapy eliminates symptoms and infection for up to 4 months. These products are biological medicines that exhibit the immunogenic components of the microbe without synthetic chemical substances through a natural and normal physiological pathway to generate an active protective immunization and cure challenging illnesses. Here, the mucosal immune system is essential because it is constantly exposed to many antigens, which activates a controlled immunological response at the local and systemic levels [104]. The efficacy of ABL was evaluated in a prospective study including 12 children with complicated UTI; five (42%) of the participants received ABL for 3 months and demonstrated ongoing improvement. After an initial negative phase, three patients (25%) had persistent UTI, whereas four patients (33%) never responded. These observations support the development of a polyvalent immunogen to treat and prevent recurring UTI [112]. Furthermore, oral ABL also stimulated bladder mucosa in the study of Ahumada-Cota et al. [111].

## 4. Potential Bacterial Lysates *In Vitro* and Preclinical Stage

### 4.1. A PMBLs of 13 Bacterial Strains

Sidoti Migliore et al. [113] examined the molecular mechanisms of bronchial epithelial cells' innate response in the presence of PMBLs containing 13 inactivated bacterial strains of common upper and lower respiratory pathogens (*S. aureus*, *S. viridans*, *S. pyogenes*, *K. pneumoniae*, *K. ozaenae*, *H. influenzae*, *M. catarrhalis*, and *S. pneumoniae*). The PMBLs increased the expression of cellular adhesion molecules such as ICAM-1, E-cadherin, and amphiregulin, a growth factor that promotes human bronchial epithelial cell proliferation. Interestingly, the PMBLs induced human bronchial epithelial cells to produce *β*-defensin-2, a potent antimicrobial peptide, resulting in direct antimicrobial action. PMBL-stimulated human bronchial epithelial cells also signal innate lymphoid cells to produce more IL-22 by innate lymphoid cells through IL-23, which may help epithelial cells release antimicrobial peptides. These findings suggest that PMBLs may improve airway epithelial cell antibacterial activity and mucosal barrier integrity [113].

### 4.2. *Mycobacterium tuberculosis* Lysates

*M. tuberculosis* (MTB)-specific antigen “early secreted antigenic target six kDa” (ESAT-6) or MTB lysate stimulated mitofusin 2 (MFN2) interaction with “nucleotide-binding domain, leucine-rich–containing family, pyrin domain–containing-3” (NLRP3) inflammasomes, resulting in inflammasome assembly and IL-1b release [114]. Further, Hirsch et al. [115] employed a crude French-press preparation of virulent MTB H37Rv with all bacterial components. MTB leads to “induced” Treg (iTreg) growth, which relies on mononuclear phagocytes expressing “transforming growth factor-beta” (TGF*β*) and “indoleamine 2,3-dioxygenase” (IDO). In this human *in vitro* model, MTB H37RvL generated CD4 + CD25hi + Foxp3+ T-regs with elevated TGF*β* expression and suppressed T-cell responses. Moreover, Crespo et al. [116] investigated whether *in vitro* MTB or *Mycobacterium leprae* lysate exposure affects *Porphyromonas gingivalis* immune responses and proposed a novel experimental immunology-paleopathology interaction. Human peripheral blood mononuclear cells (PBMC) treated with *M. leprae* or MTB lysates on day 1 show an inflammatory shift (increased TNF-*α* and IFN-*γ*) when exposed to the oral pathogen *P. gingivalis* on day 2. MTB infection or exposure to its metabolites induces a milieu rich in proinflammatory/anti-inflammatory cytokines and strong microbial activation via antigenic and TLRs components.

### 4.3. *H. influenzae* Lysate

Aerosolized *H. influenzae* lysate protected mice against respiratory infections from several pathogenic gram-positive and gram-negative bacteria and fungi, including *S. pneumoniae*, *K. pneumoniae*, *P. aeruginosa*, *Bacillus anthracis*, and *Aspergillus fumigatus* [98].

### 4.4. *S. aureus* Lysates

Camussone et al. [117] found that immunizing heifers with *S. aureus* bacterin or lysate made with ISCOM Matrix produced specific IgG in whey compared to controls. Innate immune system stimulation helps evolve adaptive immune response. This research assessed innate and T-helper activation by measuring IL-4, IL-10, IL-12p-40, TNF-*α*, and IFN-*γ* mRNA levels 24 hr after the second vaccination dosage. The vaccines increased the expression of IL-10, IL-12, and TNF-*α*. Heifers immunized with a CP5 *S. aureus* bacterin formulated with ISCOM Matrix had greater relative expression levels of IL-10 and IL-12, 24 hr after a second vaccination dose than those vaccinated with Al (OH)_3_ [118]. Both formulations showed increased activation of proinflammatory cytokines (IL-12 and TNF-*α*) and regulatory cytokines (IL-10), with early induction more obvious following vaccination with *S. aureus* lysate [117].

### 4.5. Probiotic Bacterial Lysates

An inactive bacteriologic (IAB; proprietary lysate preparation of *Lactobacillus bulgaricus*, ReseT®) and a probiotic (*Lactobacillus rhamnosus*, LGG) were tested on adult *Drosophila*. The inert lysate of *L. bulgaricus* activates a subset of conserved TLRs and “nucleotide-binding oligomerization domain-containing protein” (NOD) receptors in human cells *in vitro*. NF-*κβ* signaling patterns in the brain and abdominal tissues were changed by short-term IAB and probiotic *L. rhamnosus* therapy [119].

## 5. Bacterial Lysates or Extracts as an Immunotherapy for Cancer

Coley began treating his cancer patients with streptococcal live cultures and noticed that creating a fever was essential for tumor shrinkage; nevertheless, this method also resulted in several deaths. Consequently, Coley produced a variety of “antitumor vaccinations” by combining heat-inactivated *S. pyogenes* with *S. marcescens*, commonly known as “Coley toxins” or “Coley vaccines.” He could induce infection symptoms such as inflammation, chills, and fever without risking bacteremia [25, 26].

### 5.1. How Do BLs Combat Cancer?

“Coley toxins,” BCG, and heat-killed mycobacterial preparations may induce or modulate systemic immune activation to fight cancer. By nature, bacterial preparations stimulate innate and type 1 immunity. Most of their immune response comes from PAMPs, which interact with PRRs to activate NK, *γδ* T, and myeloid cells. Myeloid cells include M*φ*, DCs, and granulocytes, which sit between innate and adaptive immunity. DC activation leads to adaptive immunity, favoring CD4+ Th1 responses and CD8+ cytotoxic cell activity. The most significant effect of bacterial preparations may be on DCs, which are necessary for adaptive immunity because they process and present antigens to CD4+ and CD8+ lymphocytes [120, 121].

DCs fail to grow in malignancy and cannot generate type 1 responses. Hypoxia and increased synthesis of vascular endothelial growth factor (VEGF), macrophage colony-stimulating factor (M-CSF), IL-6, IDO, extracellular adenosine, and immunosuppressive cytokines, including IL-10 and TGF-*β*, characterize the cancer site's immunosuppressive milieu. All the above causes affect DC functioning in cancer to various degrees. The immunological deficiency of DCs is seen in the circulation and lymph nodes, evident in impaired systemic immune activity [122, 123]. Because bacterial products stimulate DCs, which preferentially promote Th1 development and cytotoxic immune responses, this impact may restore DC function by creating a cytokine milieu that counteracts tumor-induced immunosuppression [34].

“Coley toxins” and their recent changes may activate innate immunity, resulting in therapeutic advantages. IL-1, IL-6, IL-12, and TNF-*α* may mediate this process by creating a cytokine milieu that triggers a bacterial antigen-specific adaptive response. The cytokine milieu promoting immunological activation may restore tumor-specific Th1 and cytotoxic T cells' ability to recognize and respond to tumor antigens [124, 125].

In cancer immunology, activating and stimulating NK cells and M*φ* using bacterial products or PRRs agonists have indicated the ability to correct immunosuppressive tumor microenvironment inadequacies [126]. Classical activation of M*φ*, which is associated with high IL-12 and low IL-10, by bacterial products may offset the protumor effects of tumor-associated M*φ*, which has low IL-12 and elevated IL-10 [127, 128]. IL-10 downregulates proinflammatory cytokines, including IL-1, IL-6, and TNF-*α* effectively, although its actions are not only limited to these mediators. In fact, IL-10 also inhibits the synthesis of chemotactic substances like IL-8 and chemokines that may draw more leukocytes to the site of inflammation [129]. BCG, a nonspecific anticancer immune stimulant, causes a local inflammatory response in the bladder with infiltration of innate immune cells, including M*φ*, and lymphocytes, particularly CD4+ Th1 cells, increasing the effector/suppressor ratio [124, 125]. Type 1 immunity arises from the ensuing cytokine environment, including IL-6, IL-8, IL-10, IL-12, TNF-*α*, IFN-*γ*, granulocyte-macrophage colony-stimulating factor (GM-CSF), chemokines, and adhesion molecules [130, 131].

**Figure 2:**
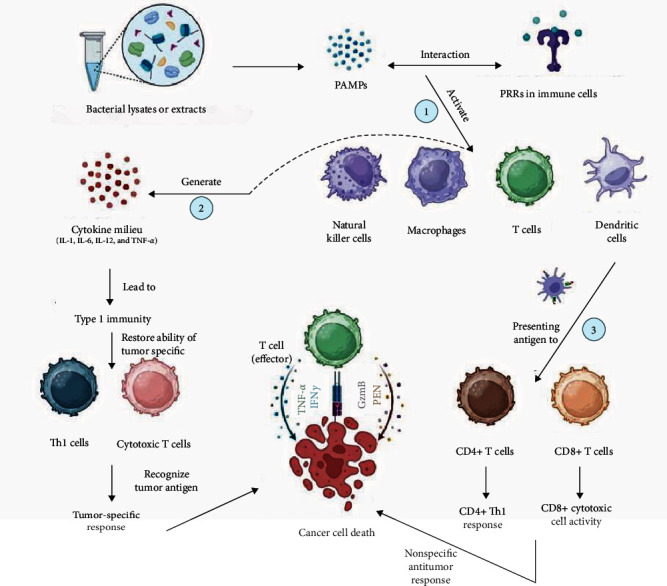
Mode of actions of bacterial lysate-based cancer immunotherapy. Pathogen recognition receptors (PRR) in immune cells such as natural killer cells, macrophages, T cells, and dendritic cells (DCs) interact with the pathogen-associated molecular pattern (PAMPs), activating the immune cells (1). This activation creates a cytokine milieu (IL-1, IL-6, IL-12, and TNF-*α*), ultimately leading to type 1 immunity. Activating type 1 immunity helps to restore the ability of tumor-specific type 1 helper T cells (Th1) and cytotoxic T cells, which then recognize cancer antigens and destroy cancer cells (2). In addition, PAMP-activated DCs gained the ability to present antigens to CD4+ T cells and CD8+ T cells. This nonspecific immune activation could also play a role in destroying cancer cells (3) (PFN, perforin; GzmB, granzyme B).

After bacterial extract treatment, vascular, ulcerating, or fungal tumors may deteriorate quickly and slough. Tumors with diminished vascularity soften and shrink. After an intravenous injection, the skin may redden, tighten, and temporarily grow in size. Immunotherapy typically causes a temporary increase in tumor growth; hence, immune-related response criteria (irRC) were developed to revise response evaluation criteria in solid tumors (RECIST) [132, 133]. A robust immune response may generate a massive inflow of immune cells into the tumor, making up to 40% of its volume. Tumors with more lymphocytes have better prognoses [134]. This influx may cause a temporary enlargement that is hard to distinguish from malignancy without a biopsy. The 12-week delay before continuing or stopping therapy is one of the main differences between RECIST and irRC. Ultimate immunotherapy responses to tumors may take up to 12 weeks. Bacterial fever specialists assess patient response based on pain reduction, blood indicator improvement, energy, mobility, and appetite. Softening indicates a response in palpable tumors [135] (Figure 2).

### 5.2. Anticancer Immune Response and PAMPs

Bacteria are immunogenic, and their highly conserved structures (such as PAMPs) could activate the innate immune system, resulting in a proinflammatory response against the tumors. PAMPs, also known as microbe-associated molecular patterns (MAMP), are one of the most effective tools to enhance tumor immune reactivity. PAMP structures are recognized by PRRs. The most significant PAMPs are bacterial DNA, which activate cGAS/STING signaling and can be detected in the cell cytoplasm; others, including unmethylated CpG motifs in bacterial DNA, activate the intracellular TLR9, lipoproteins activate TLR2, and newly generated bacterial proteins' N-formylmethionine (f-Met) stimulates f-Met receptors 1 and 2. Different bacteria have their own PAMPs that may cause inflammation in addition to these common ones [136].

In recent years, PAMP and PRR interactions have been intensively researched, indicating that they go beyond activating innate immune cells that defend against pathogens and engage in other processes, some of which are important to cancer treatment [120, 121]. The well-analyzed PRRs are TLRs, which identify bacterial lipoproteins, RNA, and DNA. PRRs include TLR-1 through TLR-12, C-type-lectin receptors (Dectin-1 and Dectin-2), and intracellular PRRs (NOD1, NOD2, NALP3, ISD, RIG-1, and MDA5) are mainly located on and inside dedicated antigen-presenting cells such DCs and M*φ* [135].

DCs are activated when they encounter antigens and PAMPs at the same moment and then migrate to the regional draining lymph nodes where they could activate antigen-specific T and B cells; besides, PAMP-stimulated DCs may develop dose-dependent NK cell helper responses [137]. To activate DCs, neither antigen nor PAMPs is enough. However, antigen-presenting DCs without PAMPs or PRRLs generate T-cell tolerance to the corresponding antigens. Conversely, properly activated T cells through costimulatory DC signals such as CD80, CD86, ICOSL, CD137, and OX40, together with DC-generated cytokines, undergo clonal expansion. Depending on the pathogen, they develop into CD8+ cytotoxic T cells (CTLs) that destroy cancer cells or Th1 and Th2. Th1 cells cause B cells to make IgG antibodies; Th2 cells induce IgM and then IgA, IgE, and IgG (humoral reaction) [138].

BCG (*in situ* bladder carcinoma), a live attenuated strain of *Mycobacterium bovis* developed initially as a tuberculosis vaccine, and Imiquimod (basal cell carcinoma) are TLRs agonists that induce TLR2 and TLR4 [139, 140] and TLR 7 [141] signaling, respectively. Both of these, and perhaps “Coley toxins” (considered as a PAMP mix), activate NF-*κ*B and transcription of proinflammatory cytokine genes such IFN-*α*, TNF-*α*, IL-6, and IL-12 through MYD88 [142, 143]. Other receptors, such as mannose, complement, and lectin, also identify microbes. Although poorly described, these receptors may induce innate and adaptive immune responses against tumors. However, certain PRRs have been linked to tumor promotion in recent years [128, 144].

When activated, several T-cell types express TLRs and may read PAMPs independently. This TLR expression is transitory and downregulated after a few days unless they receive continual PAMP stimulation [138]. In mice, B cells generating IgM, IgG1, and IgG2c express TLRs and respond to TLRs dependently, whereas IgE-producing B cells do not. In humans, similar outcomes have been discovered [145]. TLR expression on adaptive immune system cells may give additional checkpoints to detect infection [146]. These findings show that PAMPs must be continuously provided to mimic a proliferative infection. Many cancer patients have tumor-specific T cells, proving that neoplastic cells are visible to the immune system. T cells in chronic diseases may experience exhaustion, characterized by programmed cell death protein 1 (PD-1) expression and cell cycle arrest, or anergy, characterized by decreased IL-2, IFN-*γ*, and TNF-*α* expression [147]. Lack of costimulation by antigen-presenting cells (tumors do not generate PAMPs) or inhibitory stimulation by Treg cells or myeloid-derived suppressor cells (MDSC) in the tumor environment might induce anergy (tumor escape). Anergy and exhaustion may be reversed *in vivo* [148, 149]. PAMPs mediate Treg suppression of naive T cells. PAMP-activated DCs may reactivate anergic or exhausted T cells in cancer patients, with distinct T-cell clones targeting pathogens and neoplastic cells. Repeated PAMP administration in cancer mice reduces MDSC levels [150], perhaps reducing tumor escape. Orange et al. [135] suggested using combined PAMPs (multiple bacterial products) for many weeks in patients with intact immunological function. They discovered early evidence supporting this idea in mice with cancer, single PAMPs caused tumor growth to decrease, and combined PAMPs cured it [150].

### 5.3. Clinical Effectiveness of Bacterial Lysates or Extracts against Cancer

Several whole-cell BLs or extract formulations for cancer therapy have been created and explored following on from “Coley toxins.” The concept is that “Coley toxins,” mixed bacterial vaccines, BCG, heat-killed whole cell mycobacterial preparations, and pneumococcal vaccine PCV-13 improve the patient's immune system and capacity to respond to tumor cells, restricting and preventing tumor development and spread. The most effective illustration of this concept is BCG. BCG is FDA-approved for intravenous immunotherapy of *in situ* bladder cancer [29, 30]. Also, BCG is the preferred therapy for nonmuscle invasive bladder cancer because, despite its toxicity, it reduces recurrence in high-risk patients better than mitomycin C. Though sometimes used to treat melanoma, BCG has been tested in other malignancies with minimal effectiveness [30, 151]. Intralesional BCG improves survival in late-stage melanoma patients with cutaneous metastases [152]. Recently, BCG has been studied in renal and prostate cancer [30]; however, sepsis danger has restricted further development. Heat-killed formulations with comparable immunological properties but more excellent safety is considered an alternative.

Cadila Pharmaceuticals Ltd.'s heat-killed *M. indicus pranii* vaccine, FDA-approved for leprosy, has shown promising results in invasive bladder cancer, NSCLC, and advanced solid tumors refractory to standard treatments [32, 33]. This product is being evaluated in superficial transitional cell carcinomas of the bladder (NCT00694798 and NCT00694915), stage III–IV melanoma (NCT00675727), hormone-refractory metastatic prostate cancer in combination with Docetaxel (NCT00525408), and NSCLC in combination with Paclitaxel + Cisplatin (NCT00680940).

Another saprophytic nonpathogenic *Mycobacterium* studied for cancer therapy is *Mycobacterium vaccae* [153, 154]. Its favorable safety profile, immunomodulatory activities [154, 155, 156], and promising patient benefits encouraged Immodulon Therapeutics Ltd. to create a comparable product based on heat-killed whole-cell *M. obuense*. This preparation (IMM-101) demonstrated safety in a phase I clinical study (NCT01308762) in stage III/IV melanoma patients [157]. A total of 10 of 18 patients survived after almost 3 years, and seven were undergoing therapy (NCT01559818) as of September 2013 [34].

Over 30 years and hundreds of applications, Coley recorded six treatment-related deaths in his department and three from colleagues. All nine patients had incurable late-stage tumors; thus, he suspected bacterial extracts caused them. Two intravenous-injected patients died from embolism; three received an excessive initial dose, one dose that was straight into the tumor; three died from renal failure, apparently caused by tumor lysis syndrome; one received a second injection with a high fever. Thus, six of nine fatalities were avoidable. Modern therapeutic settings might successfully have treated the three supposed tumor lysis syndrome patients [135].

## 6. What Distinguishes Tumor Lysate from BLs in the Battle against Cancer?

In the context of cancer immunotherapy, BLs are generally acknowledged to be nonspecific immunostimulants, but tumor cell lysate (TCL) has the potential to function as a more targeted specific immunostimulant. A diverse array of target antigens is included in TCL, a combination of proteins generated by the induced lysis of tumor cells [158]. In addition, knowledge of important tumor-associated antigens (TAAs) and targets, including possibly undiscovered TAAs, is not a prerequisite for the TCL method of immunization [159, 160]. TCL is an attractive vaccination candidate due to its simplicity of production and storage, absence of host-specific constraints, and unnecessity of prior knowledge about particular TAAs [161]. However, it should be noted that TCL also comprises naturally present immunosuppressants in cancer cells, including hyaluronan, which is recognized for its ability to induce tolerogenic maturation of DCs and M*φ* rather than immunogenic maturation [162]. TCL is hypothesized to include agents that induce the death of immune cells, including Fas ligand and TGF*β* [163]. On the other hand, well-chosen immunostimulatory adjuvants, which are often used to stimulate DC maturation, may counterbalance the adverse effects of immunosuppressive components found in TCL [164].

Through dying, stressed, or damaged cells, several endogenous factors are translocated to the cell membrane or discharged into the extracellular environment. These signals can stimulate the immune system and commence procedures for repairing and modifying damaged tissues [165]. They are also known as damage-associated molecular patterns (DAMPs) and may serve as danger signals or adjuvants for immune cells, or they may be crucial components of homeostatic systems [166]. In the extracellular matrix of healthy tissues, DAMPs are typically nonexistent or present in extremely low concentrations. Via PRRs on their surfaces, they can interact with nearly all variants of immune cells, including DCs. TLRs, a class of membrane-spanning proteins that identify structurally conserved self- and pathogen-related chemicals, are the principal mediators of this connection [167]. There are 10 documented distinct TLRs in humans, each of which recognizes unique DAMPs [168] and PAMPs [169]. A crucial component of the innate immune response, these receptors facilitate the contact between immune cells and pathogens [170]. Induction and modulation of efficient adaptive immune responses against infections and malignancies are also significantly influenced by the signals transmitted by various TLRs [171, 172, 173, 174]. Besides, DAMP sensing has been attributed to several PRRs including receptors belonging to the family of C-type lectins, such as Mincle and CLEC9a [175], and cytosolic PRRs such as AIM, DAI, MDA-5, NLRP3, and RIG-I [166, 176].

Lysates of tumor cells are superior vehicles for delivering a vast array of antigens coupled to MHC class I/II molecules, stimulating a more comprehensive immune response [166, 177]. As indicated by the development of specific CTLs against tumor antigens and a substantial decrease in tumor size in mice models, research studies using DCs pulsed with tumor lysates have yielded noteworthy outcomes in stimulating robust immune responses [178, 179, 180, 181, 182]. Additionally, promising results have been shown in mice and humans from several trials using DCs loaded with autologous, allogeneic, and partial autologous tumor lysates [183, 184, 185, 186]. Furthermore, in several clinical trials investigating the use of allogeneic lysates derived from diverse human tumor cell lines to treat cancer, favorable outcomes have been seen [187, 188, 189, 190]. Nevertheless, immunoregulatory cytokines such as IL-10 and TGF*β* may be secreted by some cancer cells during cell culture, therefore producing a more tolerogenic phenotype on DCs, a possible obstacle rather than an advantage [166]. Several studies indicated that DC process signals from peripheral physiological or pathological microenvironments via their PRRs. The functional capacities acquired by DCs vary depending on the kind, quantity, and combinations of these stimuli [191, 192].

The use of patient-specific tumor cells to generate a tailored whole-tumor lysate (WTL) cancer vaccine would provide an extensive repertoire of known and unidentified antigenic T-cell epitopes, which are essential for eliciting a comprehensive immune response [193, 194]. These polyclonal CD8+ and CD4+ antitumor T-cell responses raised against tumor neoantigens and/or TAAs would be beneficial in reducing tumor escape variants through the loss of single or multiple antigens in the tumor cell [195, 196, 197]. The cognate assistance that CD4+ T helper cells provide to CD8+ T cells may also stimulate long-term immunological memory against the tumor. Additionally, whole-tumor vaccinations elicited a greater objective response (8.1%) in patients than specified tumor antigen-based vaccines (3.6%), according to a meta-analysis of about 1,800 patients from different cancer clinical trials [198]. The combination of heat shock-treated tumor cell lysates and *Concholepas concholepas* hemocyanin as an adjuvant in the therapeutic vaccine TRIMELVax effectively inhibits the growth of aggressive and weakly immunogenic B16F10 melanoma tumors in mice, extending their survival even in the absence of an immune checkpoint inhibitor [199]. For WTL preparation, the patient's tumor might be collected during debulking surgery. Malignancies such as glioblastomas, ovarian, breast, and lung carcinomas might potentially benefit from WTL treatment due to the ability to collect enough quantities of tumor material [200]. Allogeneic tumor cell lines that express shared TAAs may be used as a substitute when autologous tumor materials are scarce. The allogeneic melanoma tumor cell lysate Melacine (Corixa Corp.) has shown some anticancer properties when paired with the adjuvant DETOX (purified mycobacterial cell wall skeleton and monophosphoryl lipid A) [193, 201].

## 7. Future Insights

As fundamental science, technology, and clinical research advance and bacterial lysates progressively establish themselves as an innovative weapon in combating cancer and other infectious diseases, the use of BLs in these areas will probably expand. Ongoing investigations may likely reveal more mechanisms by which bacterial lysate immunotherapy operates, including how bacterial constituents control immune responses in the context of cancer and infectious diseases. This will accelerate the development of more focused medicines. Engineering bacterial lysates to improve their immunogenicity, safety, and specificity via the development of designer bacterial strains or the modification of existing strains to produce lysates with tailored therapeutic qualities might be of scientific interest [2, 37, 91].

Potential future developments in genomics and immune profiling technologies might facilitate the identification of blood-based immune signals that can be used to predict a patient's response to bacterial lysate immunotherapy under individualized treatment regimens. Subsequent investigations may concentrate on ascertaining if bacterial lysates may be used to create a tumor microenvironment that is more conducive to antitumor immune responses [40, 192, 202].

Further research may investigate other combination techniques that combine bacterial lysate immunotherapy with checkpoint inhibitors, chemotherapy, or targeted treatments. Combinatorial medicines are expected to boost treatment efficacies, circumvent resistance mechanisms, and enhance anticancer or anti-infection immune responses. Therapeutic studies will be required in the future to confirm the safety, effectiveness, and clinical value of bacterial lysate immunotherapy in diverse patient populations [193, 203].

## 8. Conclusions

A strong interplay between specific (acquired) and nonspecific (natural, innate) immune mechanisms plays a crucial role in protecting against infectious agents. A nonspecific response actively supports specific response mechanisms. This enables effective protection of our body against infections, both at the stage when pathogens reach the body and after their penetration into tissues. Stimulating microbial antigens such as bacterial lysates (BLs) or extracts activates specific immunity mechanisms: humoral and cell-mediated responses and nonspecific immunity. Specific antibodies or immunoglobulins of various classes are involved in the humoral response to protect from recurrent infections. BLs have been found effective in children and adults in treating respiratory tract infections, chronic obstructive pulmonary diseases, rhinitis, rhinosinusitis, and urinary tract infections. The World Health Organization and the Centers for Disease Control have declared antibiotic resistance as one of the most significant risks to humanity in our lifetime. There is a pressing need to utilize new antibiotic-free therapies (such as BLs) in preventing UTI recurrences. Correspondingly, BL-based nonspecific immune stimulants could help the immune system detect and fight against cancers such as sarcomas, carcinomas, lymphomas, melanomas, myelomas, bladder cancer, and non-small cell lung cancer. The late stage of cancer that is sensitive to conventional therapies becomes resistant later. Biological therapies such as BLs or extracts-based preparations are helpful to fight tumor cells in addition to traditional therapies because they are characterized by less toxicity and specific targeting of tumor cells. There is an urgent need for high-quality, large-sample-size studies on the clinical efficacy and mechanisms of action of BLs with various bacterial antigen compositions, methods of preparation, and routes of administration to determine their potential for treating infectious diseases and cancer as well.

## Figures and Tables

**Table 1:** Commercially available immunotherapeutic bacterial lysates or extract-based preparations effectively manage infectious diseases.

Bacterial lysates	Study type	Immune response and therapeutic significance	Host	References
OM−85 (*H. influenzae*, *Diplococcus pneumoniae*, *K. pneumoniae*, *K. ozaenae*, *S. aureus*, *S. pyogenes*, *Streptococcus viridans*, and *Neisseria catarrhalis*)	Clinical trial of 108 children: standard therapy (57) and standard therapy + OM-85 (51)	(a) Normalized the phagocytic level; concentration of CD3+, CD4+, and CD21+ lymphocytes; and restore the IgA and IgM levels (b) Reduced the period of acute intoxication and the likelihood of bacterial complications in respiratory tract infections (RTIs)	Children (1–3 years)	[52]
	Meta-analysis (13 RCTs)	Significantly reduced the mean number of chronic obstructive pulmonary disease (COPD) exacerbations (*p* < 0.01; WMD = −0.86; 95% confidence interval CI (−1.38, −0.34)) and the days of antibiotic treatment (*p* < 0.01; WMD = −9.49; 95% CI (−11.93, −7.05))	Adults (20–82 years)	[59]
	Double-blind, randomized control trial (RCT) (*N* = 60)	(a) Increased IFN-*γ*, decreased IL-4 and IL-13 in nasal lavage, and decreased eosinophils in nasal swabs (b) In acute allergic rhinitis (AR), decreased total nasal symptom (*p* < 0.05), itching (*p* < 0.05), nasal rhinorrhea (*p* < 0.05), and sneezing (*p* < 0.05) scores	Adults (mean age 31.34 years: OM-85 group 33.34 ± 3.21 years and placebo group 29.33 ± 4.13 years)	[60]
	Meta-analysis (53 studies)	Reduced the frequency of RTIs (MD = −2.33, 95% CI (−2.75, −1.90), *p* < 0.001)	Children (1–16 years)	[13]
	RCT	Reduced the frequency of rhinosinusitis episodes	Children (4–12 years)	[22]
	Clinical study	A 20% reduction in the occurrence of repeated events of atopic dermatitis (AD)	Children (0.5–7 years)	[62]
	Open-label, prospective, sequential study (*N* = 84)	(a) Increased serum and salivary secretory Ig (b) Reduce of the number RTIs, exacerbations of AR, asthma, or COPD	Children and adults (16–65 years)	[51]
	Clinical trial (*N* = 60)	(a) Numbers of peripheral blood NKT cells and CD4+ NKT cells were higher, and IFN-*γ*, IL-4, and IL-10 levels were increased (b) Prevented recurrent RTIs and reduced asthma attack severity	Children (5–14 years)	[57]
	Randomized, double-blind, placebo-controlled clinical trial (*N* = 428)	Reduced chronic bronchitis and COPD significantly	Adults (40–75 years)	[58]
	Prospective, randomized, single-blind study (*N* = 33)	Decreased respiratory morbidity (*p* < 0.05)	Children (3–5 years)	[53]
	Randomized placebo-controlled trial (*N* = 606)	Treatment with OM-85 has no impact on AD prevalence after 3 years of follow-up; however, reduced AD occurrence in infants with a single atopic family history	Newborns (4–5 weeks)	[54]
	Double-blind, placebo-controlled RCT (*N* = 80)	Nasal colonization of *S. aureus* has not been affected by the sublingual treatment in children with seasonal AR	Children (5–17 years)	[70]
	Double-blind, placebo-controlled, multicenter RCT (*N* = 49)	Have a preventative impact on episodes of asthma exacerbation connected to infections as well as those unrelated to them	Children	[71]
Ismigen (a PMBL of *S. aureus*, *S. pyogenes*, *S. viridans*, *S. pneumoniae* (six different serotypes: TY I/EQII, TY2/EQ22, TY3/EQ 14, TY5/EQ 15, TY8/EQ23, and TY47/EQ24), *K. pneumoniae*, K. ozaenae, *H. influenzae* serotype B, and *Moraxella catharralis*)	Double-blind RCT (*N* = 70)	(a) Reduced eosinophils in nasal swabs (b) Decreased overall nasal symptom score (*p*=0.01) and nasal visual analog scale (*p* < 0.001); increased peak nasal inspiratory flow (*p*=0.04)	Children (5–17 years, mean age: 9.23 years)	[15]
	Clinical trial (*N* = 31)	In patients with chronic decompensated tonsillitis, selectively antagonistic activity against the most common respiratory pathogens (*S. aureus*, *S. pyogenes*, *K. pneumoniae*, *H. influenzae*, and *M. catarrhalis*)	—	[72]
	A phase IV, randomized, controlled, double-blind, multicenter AIACE (Advanced Immunological Approach in COPD Exacerbation) trial (*N* = 49)	Have no impact on the reduction in exacerbations of COPD	Children	[73]
	Meta-analysis (15 RCTs, *N* = 2,557) (this study is for all PMBL)	Reduced the recurrence of RTIs (relative risk: −0.513, 95% CI (−0.722 − −0.303), *p*=0.001)	Children and adults	[8]
	Double-blind RCT (*N* = 41)	(a) Increased serum IL-4 (b) Reduced (61.5%) symptoms severity of AR	Children and adults (5–78 years, mean age 28.9 years)	[21]
	Double-blind, randomized, placebo-controlled trial (*N* = 178)	Reduced frequency (215 vs. 248) and length (10.6 vs. 15.8 days) of exacerbations and decreased antibiotic usage (−270 doses) and hospitalization time (275 vs. 590 days) in patients with moderate to severe COPD	—	[14]

Lantigen B (a PCBL of *S. aureus*, *K. pneumoniae*, *S. pneumoniae*, *S. pyogenes*, *M. catarrhalis*, and *H. influenzae*)	Double-blind, placebo-controlled, multicenter clinical study (*N* = 160)	Reduced episodes of recurrent RTIs (95% CI (1.01, 1.86))	Adults (18–65 years)	[64]

LW 50020 (Luivac) (*S. aureus*, *S. mitis*, *S. pyogenes*, *S. pneumoniae*, *K. pneumoniae*, *M. catarrhalis*, and *H. influenzae*)	Open-label, non-comparative study (*N* = 33)	Prevented RTIs	Adults (mean age: 34.0 ± 14.7 years)	[74]
	Multicenter, open study (*N* = 4965)	Decreased the frequency, severity, and duration of RTIs and their socioeconomic costs	Children and adults (3–86 years; mean, 19 years; median, 11 years)	[75]

Polyvaccinum mite (a PCBL consists of *S. aureus*, *S. epidermidis*, *S. salivarius*, *S. pneumoniae*, *S. pyogenes*, *E. coli*, *K. pneumoniae*, *H. influenzae*, *C. pseudodiphtheriticum*, and *M. catarrhalis*)	Double-blind RCT (*N* = 40)	Increased peak nasal inspiratory flow (*p*=0.01) and decreased visual analog scale for nasal symptoms (*p*=0.03)	Children (mean age, 8.7 years)	[76]

OM-89 (Uro-Vaxom) (bacterial extracts from 18 uropathogenic *E. coli* strains)	Retrospective review of medical records (*N* = 52)	Effective treatment for the management of recurrent cystitis in female	Adult women (54.4 ± 12.6 years)	[77]
	Multinational, randomized double-blinded placebo-controlled trial (*N* = 453)	Decreased mean rate of urinary tract infections (UTIs) (*p* < 0.003)	Adult women (18–65 years)	[78]
	Open-label, multicentre pilot study (*N* = 62)	Reduced incidence UTIs (*p =* 0.001)	Women of 16–28 weeks of pregnancy (age not found)	[79]
	Randomized multicenter double-blind trial (*N* = 112)	Decreased the number of recurrences (*p* < 0.001) of UTIs	Adult (details not found)	[80]
	Clinical study (*N* = 74)	Decreased the incidence of reinfection in patients with lower UTIs	Adult women (18–77 years, means 37.47 years)	[81]
	Double-blind cross-over study (*N* = 70)	Reduced UTIs recurrence rate	Female (13–80 years)	[82]
	Clinical study (*N* = 521) (women, 365; and men, 86)	Reduced number of recurrences of UTI (*p* < 0.001)	Adults (51.8 ± 0.9 years)	[83]

Uromune (an inactivated bacterial cell suspension of selected strains of *E. coli*, *K. pneumoniae*, *P. vulgaris*, and *E. faecalis*)	Prospective study (*N* = 77)	Reduced recurrent UTIs	Adult women (18–87 years, mean age 56 years)	[84]
	Retrospective cohort study evaluating the medical records (*N* = 669)	Reduced the incidence of recurrent UTIs and antibiotic consumption	Female (age not known)	[85]
	Multicentred observational study (*N* = 319)	Reduced recurrent UTIs	Adult women (18–87 years)	[86]

Urovac (a mixture of heat-killed 10 uropathogenic strains, including 6 *E. coli* strains and 1 strain each of *P. mirabilis*, *P. morganii*, *E. faecalis*, and *K. pneumoniae* in equal proportions)	Randomized, double-blind, placebo-controlled clinical trial (*N* = 75)	Reduced recurrence of UTIs	Adult women (19–70 years)	[87]
	Double-blind, placebo-controlled, phase 2 clinical trial (*N* = 54)	Helped to remain infection-free	Adult women (18−74 years)	[88]
	Randomized, double-blind, placebo-controlled clinical trial (*N* = 91)	Delayed the interval in reinfection of recurrent UTIs	Adult women (18–82 years)	[89]

Urivac (lysates of *P. acnes*, *K. pneumoniae*, *P. aeruginosa*, *E. faecalis*, *E. coli*, *P. mirabilis*)	Prospective, open, multicenter, parallel-group randomized CRUTIL study (*N* = 83)	87% (19) relapse-free patients who had received the vaccine	Children (3–15 years)	[16]
ExPEC4V (had four bioconjugates with the O-antigens of the four extraintestinal pathogenic *E coli* serotypes: O1A, O2, O6A, and O25B.)	Randomized, placebo-controlled single-blind (*N* = 196)	Reduced UTIs	Adult women (18–70 years)	[90]

*Note*: AR, allergic rhinitis; CI, confidence interval; COPD, chronic obstructive pulmonary disease; Ig, Immunoglobulin; MD, mean difference; N, subject numbers (sample size); PMBL, polymicrobial bacterial lysate; RCT, randomized control trial; RTIs, respiratory tract infections; UTIs, urinary tract infections; WMD, weighted mean difference.

## Abbreviations

AD: Atopic dermatitis
ALIs: Air–liquid interfaces
APRIL: A proliferation-inducing ligand
AR: Allergic rhinitis
BAFF: B-cell-activating factor belonging to the tumor necrosis factor (TNF) family
BCG: Bacillus Calmette– Guérin
BLs: Bacterial lysates
CBF: Ciliary beat frequency
CCL: Chemokine (C-C motif) ligand
CD: Cluster of differentiation
cGAS: Cyclic GMP-AMP synthase
CI: Confidence interval
COPD: Chronic obstructive pulmonary disease
CpG: Unmethylated cytosine–guanine dinucleotide
CRS: Chronic rhinosinusitis
DAMPs: Damage-associated molecular patterns
ESAT-6: Early secreted antigenic target 6 kDa
f-Met: N-formylmethionine
GM-CSF: Granulocyte-macrophage colony-stimulating factor
HR: Hazard ratio
IAB: Inactive bacteriologic
ICAM-1: Intracellular adhesion molecule 1
ICOSL: Inducible T-cell costimulator ligand
ICS: Inhaled corticosteroid
IDO: Indoleamine 2,3-dioxygenase
IFN: Interferons
Ig: Immunoglobulins
IL: Interleukins
irRC: Immune-related response criteria
iTreg: Induced regulatory T cells
MAMP: Microbe-associated molecular patterns
MAPK: Mitogen-activated protein kinase
M-CSF: Macrophage colony-stimulating factor
MD: Mean difference
MDSC: Myeloid-derived suppressor cells
MFN2: Mitofusin 2
mL: Milliliter
Myd88: Myeloid differentiation primary response 8
MyD88: Myeloid differentiation primary response 88
M*φ*: Macrophages
NALP3: NACHT, leucine-rich region, and PYD domain–containing protein-3
NF-kB: Nuclear factor kappa-light-chain-enhancer of activated B cells
NK: Natural killer cells
NKT: Natural killer T cells
NLRP3: Nucleotide-binding domain, leucine-rich–containing family, pyrin domain–containing-3
NO: Nitric oxide
NOD: Nucleotide-binding oligomerization domain-containing protein
NOS: Nitric oxide synthase
NSCLC: Non-small cell lung cancers
PAMPs: Pathogen-associated molecular patterns
PBMC: Peripheral blood mononuclear cells
PCBLs: Polyvalent chemical bacterial lysates
PD-1: Programmed cell death protein 1
PLC*β*2: Phospholipase C isoform *β*2
PMBLs: Polyvalent mechanical bacterial lysates
PRRs: Pattern recognition receptors
RECIST: Response evaluation criteria in solid tumors
RR: Risk ratio
RRTIs: Recurrent respiratory tract infections
RTIs: Respiratory tract infections
SARS-CoV-2: Severe acute respiratory syndrome coronavirus 2
sIgA: Secretory IgA
STING: Stimulator of interferon genes
T2R: Bitter taste receptors
TAAs: Tumor-associated antigens
TCL: Tumor cell lysate
TGF*β*: Transforming growth factor-beta
Th1: Type 1 T helper cells
Th2: Type 2 T helper cells
TLRs: Toll-like receptors
TNF-*α*: Tumor necrosis factor *α*
Treg: Regulatory T cells
Trif: TIR (Toll/interleukin-1 receptor) domain-containing adaptor protein inducing interferon *β*
TRPM5: Transient receptor potential cation channel subfamily M member 5
UTIs: Urinary tract infections
VEGF: Vascular endothelial growth factor
WTL: Whole-tumor lysate
WMD: Weighted mean difference.
